# Conservative Management of Invasive Group A Streptococcal Infection With Cervical Necrotizing Fasciitis-Spectrum Disease and Pneumonia in a Healthy Young Adult

**DOI:** 10.7759/cureus.112338

**Published:** 2026-07-09

**Authors:** Phannoch Neun, Kenji Iwai, Nobuichiro Yagi

**Affiliations:** 1 Internal Medicine Department, Sunrise Japan Hospital Phnom Penh, Phnom Penh, KHM; 2 Pediatric Department, Sunrise Japan Hospital Phnom Penh, Phnom Penh, KHM

**Keywords:** clindamycin, invasive group a streptococcus, necrotizing fasciitis, non-surgical management, pneumomediastinum

## Abstract

Invasive Group A streptococcal (iGAS) infections are life-threatening conditions classically manifesting as necrotizing fasciitis or severe pneumonia, but their simultaneous occurrence in a previously healthy adult is extremely rare. While necrotizing fasciitis typically mandates urgent surgery, this report highlights a case of concurrent cervical necrotizing fasciitis-spectrum disease and pneumonia successfully managed through a conservative, non-surgical approach. A 29-year-old previously healthy Japanese male presented with a two-day history of fever, sore throat, and a maculopapular rash, with a throat swab positive for GAS. On Day 2, he acutely developed progressive dyspnea, hypoxemia, and extensive cervical subcutaneous emphysema. Computed tomography confirmed diffuse fascial edema and bilateral pulmonary infiltrates without signs of gas-forming abscess or irreversible tissue necrosis. Despite early amoxicillin administration, blood cultures remained negative, suggesting toxin-mediated clinical deterioration. Based on the imaging findings and close clinical monitoring, a non-operative strategy was selected. This was achieved by immediate medical escalation to broad-spectrum antimicrobials, including clindamycin to suppress toxin production, alongside meropenem and high-dose penicillin G. The patient recovered fully without surgical intervention. This case highlights that iGAS can cause rapid, concurrent soft-tissue and pulmonary involvement even in healthy individuals. In select cases of necrotizing fasciitis-spectrum disease where irreversible tissue necrosis is absent on prompt imaging and close clinical monitoring is maintained, aggressive multi-drug medical escalation incorporating antitoxin therapy can lead to successful outcomes without the need for morbid surgical procedures.

## Introduction

Invasive Group A streptococcal (iGAS) infection, caused by *Streptococcus pyogenes*, is a potentially life-threatening condition classically associated with severe soft-tissue and systemic diseases, including necrotizing fasciitis, streptococcal toxic shock syndrome, and severe pneumonia [1]. The global burden of iGAS remains substantial, with an increasing incidence reported across several regions [2-4]. Necrotizing fasciitis represents one of the most severe manifestations and is characterized by rapid tissue destruction, systemic toxicity, and high mortality rates; it typically necessitates urgent surgical debridement in conjunction with antimicrobial therapy [5]. Pneumonia caused by GAS, while uncommon, is associated with rapid clinical deterioration and poor outcomes [6]; the simultaneous occurrence of necrotizing fasciitis and GAS pneumonia has rarely been reported. Furthermore, invasive manifestations are more frequently observed in patients with underlying comorbidities [7,8]. In this case report, we present a rare case of iGAS infection in a previously healthy 29-year-old man presenting with concurrent cervical necrotizing fasciitis-spectrum disease and pneumonia. Notably, because imaging showed no irreversible tissue necrosis and the patient remained hemodynamically stable, a conservative management strategy was employed, resulting in rapid clinical improvement without the need for surgical intervention.

## Case presentation

A previously healthy 29-year-old Japanese male presented to the emergency department with a two-day history of fever, sore throat, and a generalized maculopapular rash involving the face and neck. He denied any recent trauma, illicit drug use, or known sick contacts. Upon arrival, his vital signs were as follows: blood pressure of 111/75 mmHg, pulse rate of 95 beats/min, respiratory rate of 24 breaths/min, body temperature of 39.1 °C, and oxygen saturation of 91% on room air. Physical examination revealed an erythematous maculopapular rash predominantly on the face that blanched upon pressure, with sparser lesions noted on the trunk and extremities. Bilateral conjunctival injection was present. Crucially, local examination of the neck at presentation revealed swelling and exquisite tenderness, but there was no palpable crepitus, skin discoloration, fluctuance, or bullae. Furthermore, despite mild tachycardia and tachypnea, the patient showed no signs of overt septic shock, maintaining a stable blood pressure without fluid resuscitation or vasopressor support. Chest auscultation initially revealed clear lung sounds.

Initial laboratory investigations revealed elevated inflammatory markers, with a white blood cell count of 9,560/μL and C-reactive protein of 9.20 mg/dL. Marked hepatic dysfunction was present, characterized by aspartate aminotransferase (AST) of 815 U/L, alanine transaminase (ALT) of 679 U/L, and gamma-glutamyl transferase (GGT) of 168 U/L. A rapid antigen detection test (RADT) performed on a throat swab was positive for GAS (Table 1). Standard blood cultures were drawn prior to the escalation of therapy; however, they ultimately yielded no growth, potentially due to the rapid initiation of antibiotics or a predominantly toxin-mediated clinical course. Despite the significant transaminitis and signs of systemic illness, oral amoxicillin was initially chosen because the patient was initially suspected to have severe uncomplicated streptococcal pharyngitis with an associated reactive rash, and the severe acute respiratory distress and rapid cervical swelling did not fully manifest and deteriorate until day two of presentation.

**Table 1:** Initial laboratory investigations upon admission.

Laboratory Parameter	Patient Result	Reference Range and Units	Status
White blood cell (WBC) count	9,560	3900-9800 /μL	Normal
C-reactive protein (CRP)	9.20	0-0.3 mg/dL	Elevated
Aspartate aminotransferase (AST)	815	15-37 U/L	Elevated
Alanine aminotransferase (ALT)	679	16-63 U/L	Elevated
Gamma-glutamyl transferase (GGT)	168	15-85 U/L	Elevated
Rapid antigen detection test (RADT) for GAS	Positive	Negative	Abnormal

**Figure 1:**
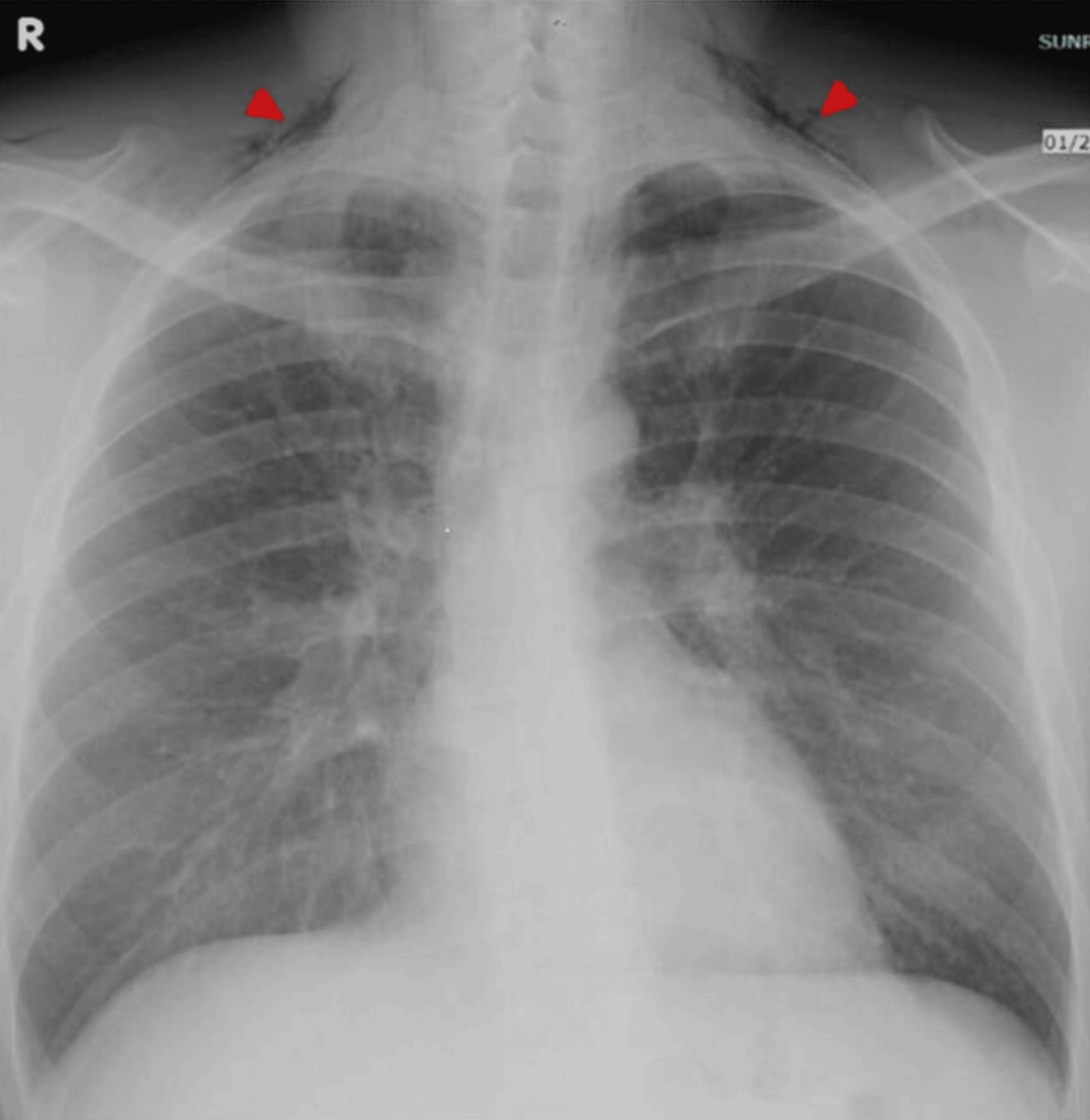
Chest X-ray revealed free air at the cervicothoracic junction (red arrows).

The patient was subsequently admitted for further evaluation and monitoring, and empiric oral amoxicillin was initiated. On hospital Day 2, the patient developed progressive dyspnea, hypoxemia, and extensive subcutaneous emphysema extending from the cervical region to the anterior chest wall. A chest radiograph revealed a pneumomediastinum at the cervicothoracic junction that was absent on admission (Figure 1).

**Figure 2:**
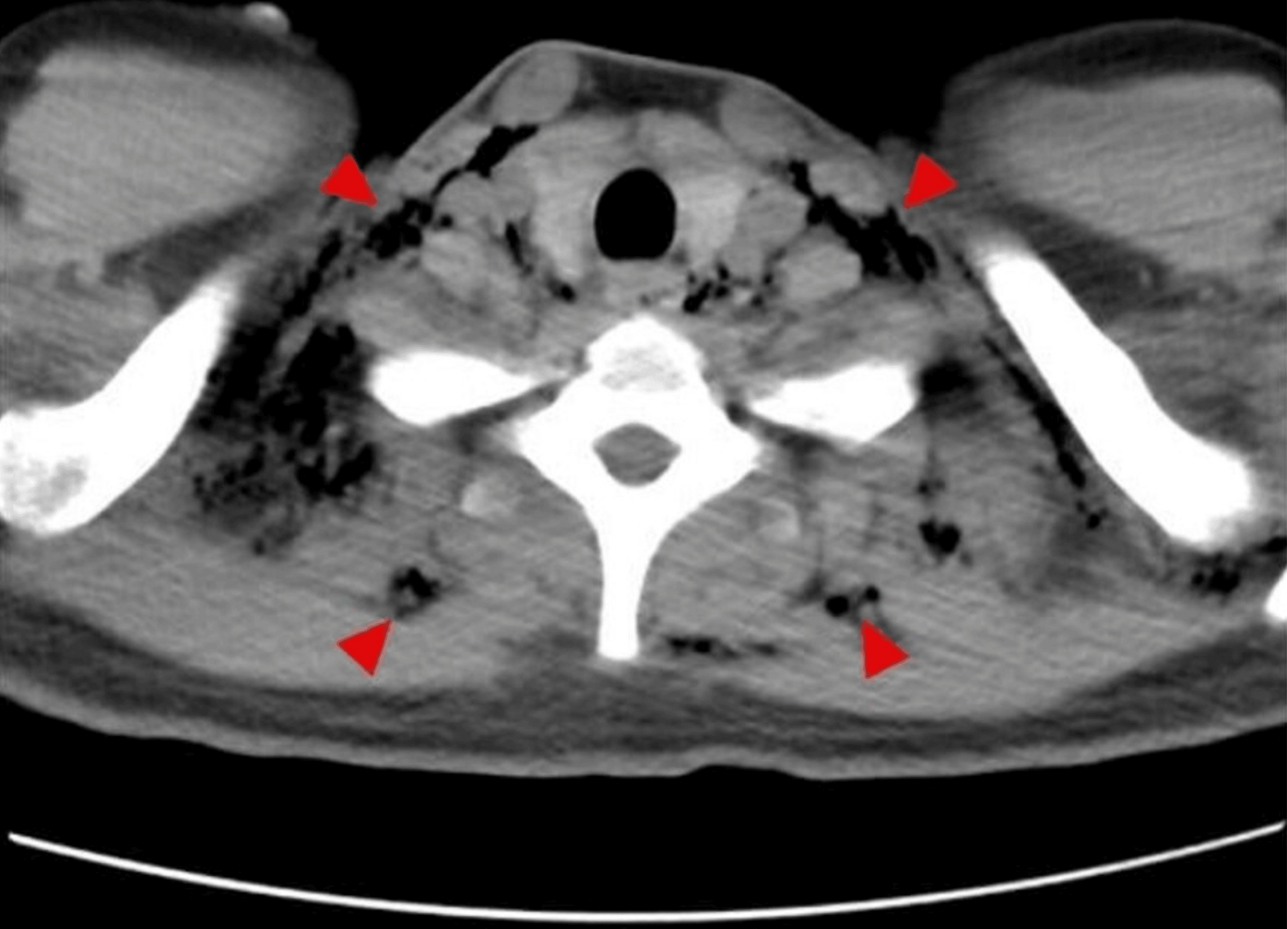
Contrast-enhanced CT showed extensive subcutaneous emphysema from the cervical to mediastinal regions (red arrows).

Contrast-enhanced computed tomography (CT) of the neck and chest confirmed extensive subcutaneous emphysema involving the cervical and mediastinal regions, as well as diffuse fascial edema without evidence of abscess formation or fluid collection (Figure 2).

**Figure 3:**
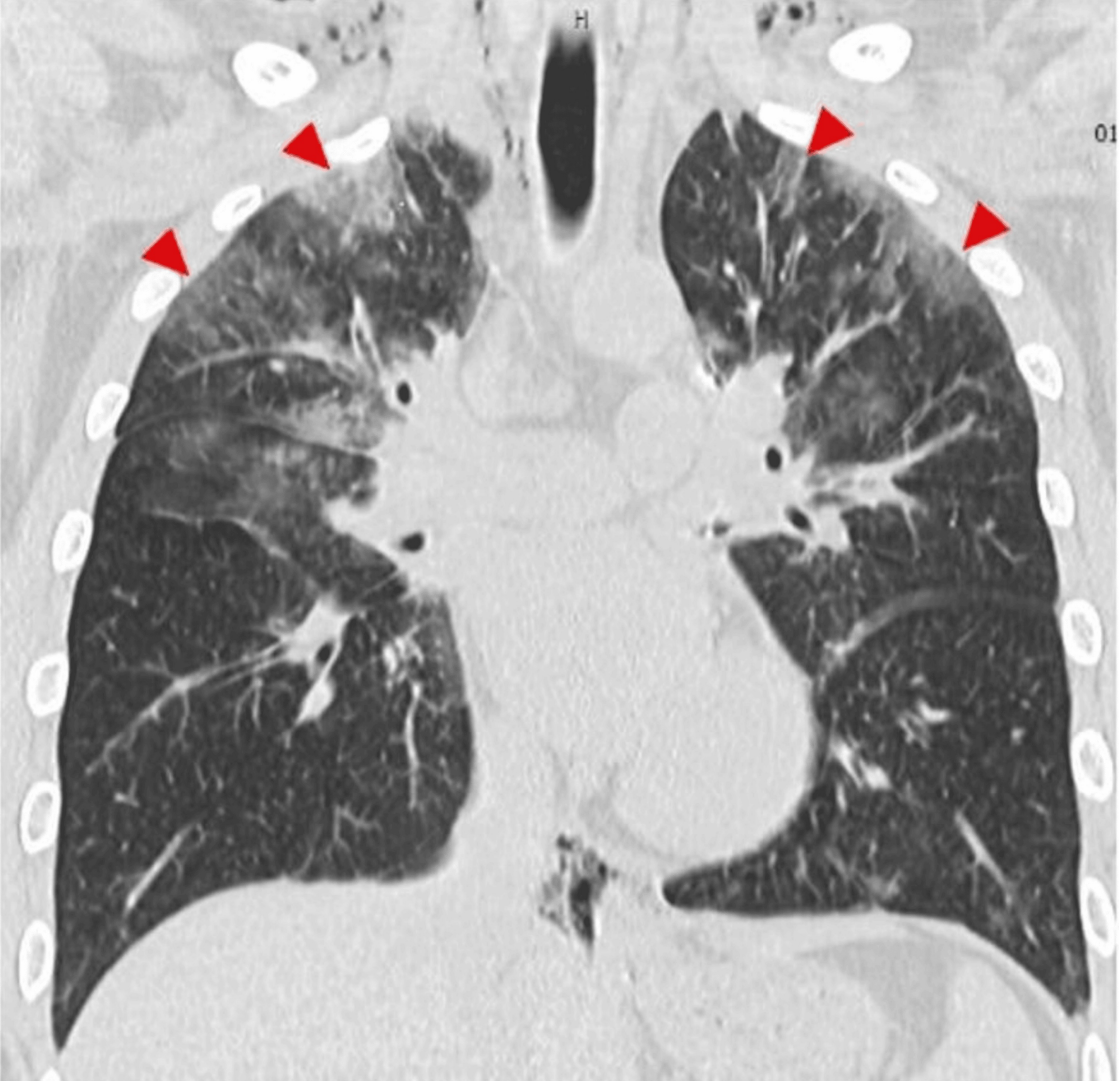
Contrast-enhanced CT showed bilateral apical infiltrates without abscess formation (red arrows).

Additionally, CT findings revealed bilateral apical pulmonary infiltrates consistent with pneumonia. No evidence of tracheal or esophageal injury was identified (Figure 3).

**Figure 4:**
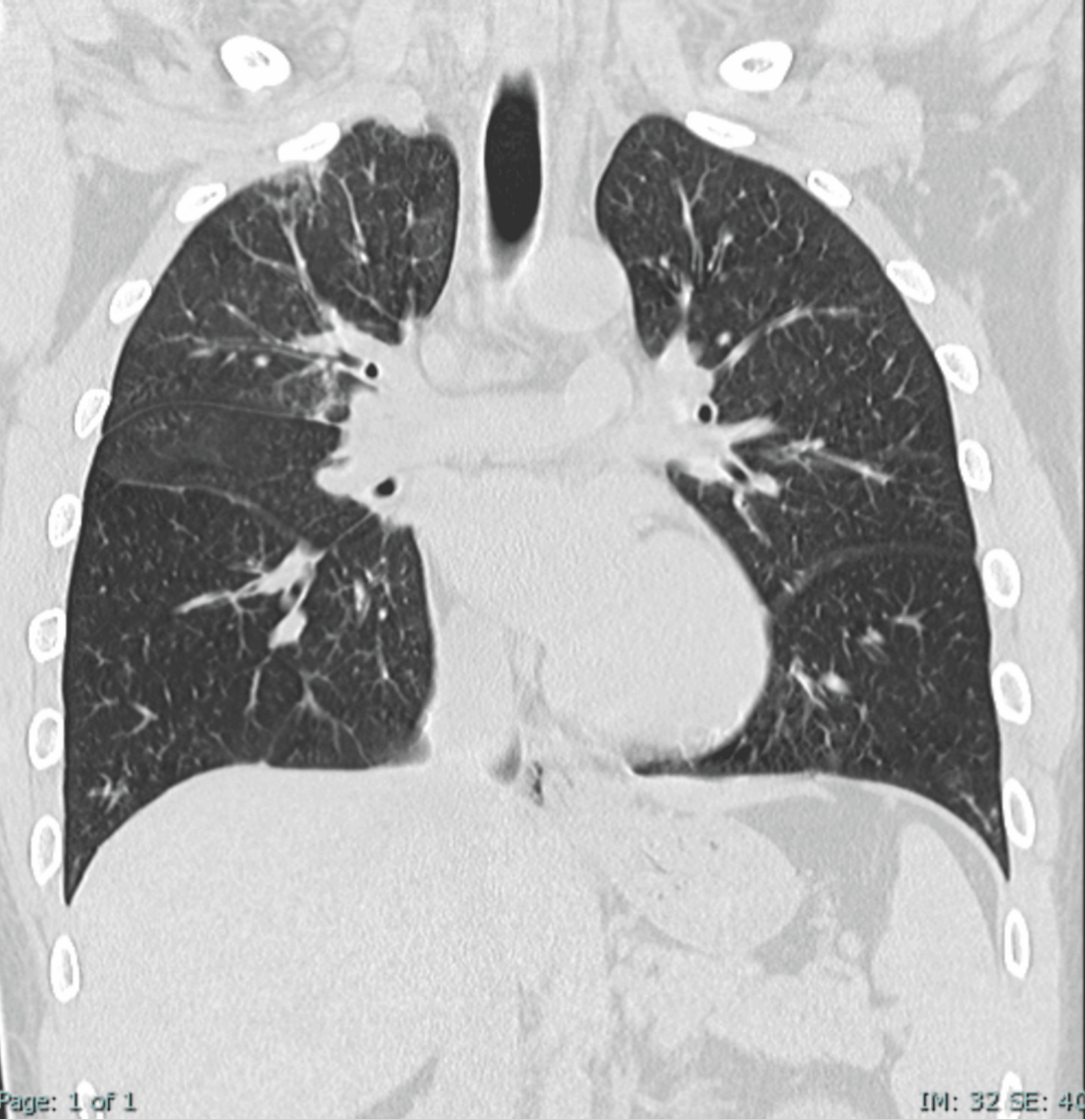
Follow-up chest computed tomography (CT) on hospital Day 10 demonstrating marked resolution of the bilateral pulmonary infiltrates.

**Figure 5:**
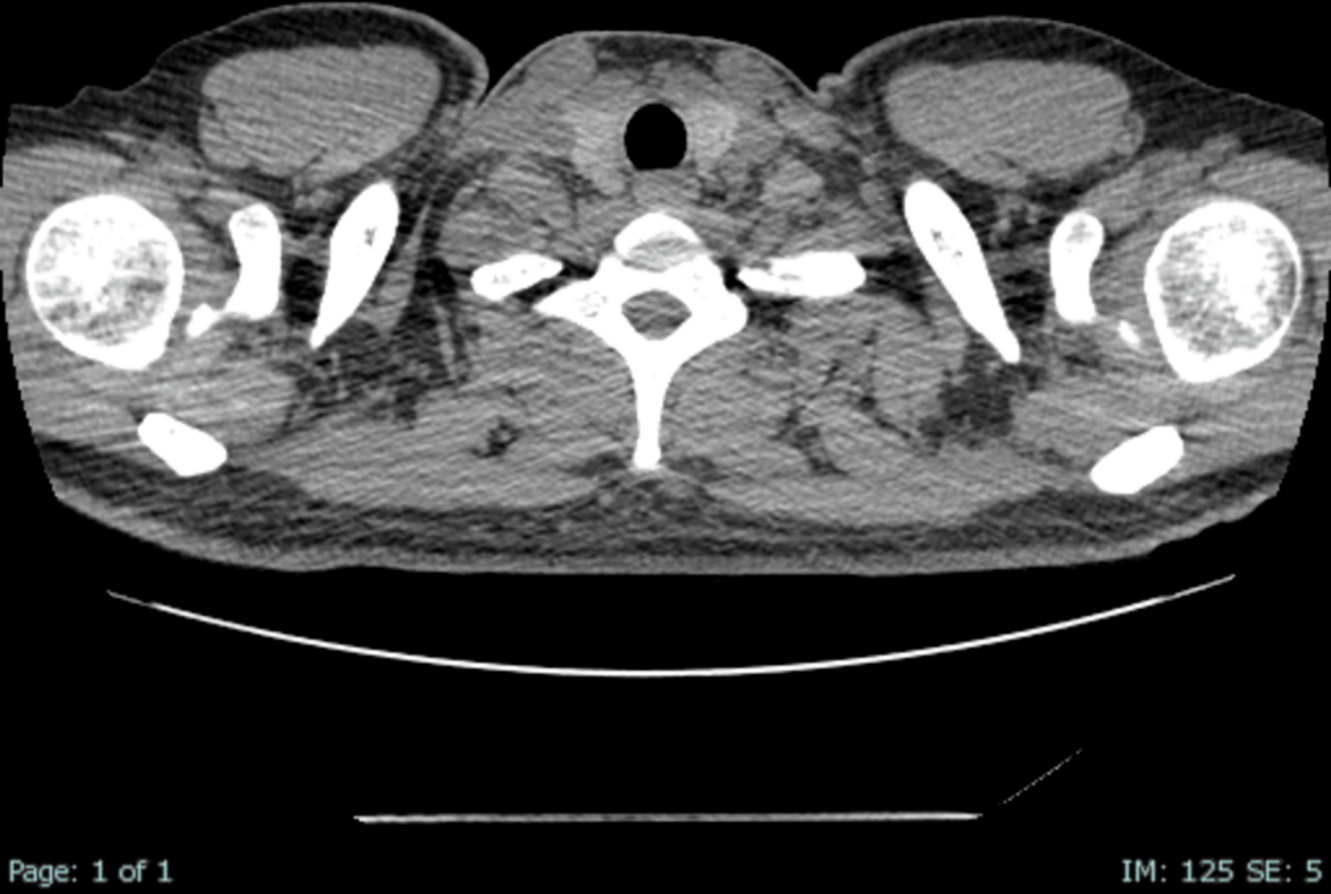
Follow-up chest computed tomography (CT) on hospital Day 10 demonstrating marked resolution of the subcutaneous emphysema.

Based on the rapid clinical progression and imaging findings, the patient was diagnosed with iGAS infection complicated by necrotizing fasciitis-spectrum disease and pneumonia. A surgical consultation was obtained; however, given the absence of radiologic or clinical evidence of frank necrosis or abscess formation, a conservative, non-surgical management strategy was pursued. Broad-spectrum antimicrobial therapy was promptly initiated with intravenous meropenem (1 g every eight hours), clindamycin (900 mg every eight hours), and high-dose penicillin G (4 million units every four hours). The patient's respiratory status gradually improved in parallel with the resolution of systemic inflammation, hepatic dysfunction, and cutaneous manifestations. Blood cultures obtained at multiple time points remained negative. A follow-up chest CT on hospital Day 10 demonstrated marked resolution of the bilateral pulmonary infiltrates and subcutaneous emphysema (Figures 4-5).

Subsequently, magnetic resonance imaging (MRI) on Day 12 confirmed the absence of abscess formation or progressive fascial disease, facilitating the de-escalation to oral antibiotic therapy. The patient was transitioned to oral amoxicillin hydrate/potassium clavulanate (500 mg/125 mg) and was discharged in stable condition on hospital Day 14 with complete clinical recovery.

## Discussion

We present a case of iGAS infection in a previously healthy young patient who developed concomitant necrotizing fasciitis-spectrum disease and pneumonia, achieving complete recovery without surgical intervention. This case highlights two pivotal clinical observations. First, iGAS infection can manifest with simultaneous necrotizing fasciitis-spectrum involvement and pneumonia, even in patients lacking traditional risk factors or comorbidities. Second, surgical intervention may potentially be avoided through early clinical recognition and the prompt initiation of aggressive antimicrobial therapy. Regarding the first point, iGAS infection is a well-recognized cause of severe, life-threatening conditions, including necrotizing fasciitis, pneumonia, and streptococcal toxic shock syndrome (STSS). However, the concomitant occurrence of necrotizing fasciitis and pneumonia in a patient without pre-existing comorbidities has rarely been documented in the literature. iGAS can cause a variety of complications, including necrotizing soft-tissue infections (NSTIs), bacteremia, and pneumonia [9]. Necrotizing fasciitis can be caused by several different pathogens; however, GAS is one of the most common etiologic agents, particularly in type II necrotizing fasciitis, which is typically monomicrobial. In such cases, the infection often originates in deep tissues without a clear portal of entry [10]. In terms of pneumonia caused by iGAS, cases have been reported in previously healthy individuals in the past [11]; however, *S. pyogenes* remains an uncommon pathogen for community-acquired pneumonia (CAP), and it is associated with a high mortality rate [6,12]. In the absence of surgical exploration or histopathological confirmation, several alternative diagnoses within the GAS-associated soft-tissue infection spectrum must be considered. These include severe cervical cellulitis, a deep neck space infection, or non-necrotizing fasciitis. Distinguishing between these entities clinically can be exceedingly difficult. While severe cellulitis involves the dermis and subcutaneous tissues, the presence of progressive subcutaneous emphysema and diffuse fascial edema on CT strongly pointed toward a deeper process affecting the fascial planes. While a deep neck space infection or non-necrotizing deep tissue infection could fully explain the clinical picture without true fascial necrosis, the rapid clinical deterioration necessitated a treatment strategy calibrated against the most dangerous entity on this spectrum-necrotizing fasciitis - even as the ultimate lack of irreversible necrosis allowed for successful non-operative management. In this case, despite the concomitant presentation of two aggressive clinical entities, we were unable to isolate the pathogen from multiple blood cultures. These negative results likely resulted from the prior administration of amoxicillin (AMPC). While AMPC may have reduced the viable bacterial load below the detection threshold, it could not neutralize pre-existing exotoxins and superantigens. This created a clinical paradox in which the patient's condition deteriorated due to toxin-mediated systemic inflammation and hematogenous spread, even as the antibiotics were effectively sterilizing the bloodstream [13].

Previous research has similarly reported that the positivity rate of blood cultures in GAS-associated necrotizing fasciitis is only 11%-60% [14]. This underscores the critical importance of integrating clinical findings, RADTs, and imaging studies when evaluating suspected iGAS infection. In addition to this unusual simultaneous presentation, the patient lacked previously reported risk factors, such as intravenous drug use, HIV infection, diabetes mellitus, trauma, skin lesions, or preceding viral infections [7,8,15]. Thus, clinicians must remain vigilant, as iGAS infections can manifest in previously healthy adults without any underlying comorbidities. As demonstrated in this case, iGAS can trigger precipitous clinical deterioration, leading to the concurrent development of pneumonia and necrotizing fasciitis-spectrum disease. Regarding the second point, although early surgical debridement is generally considered the standard of care for definitive necrotizing fasciitis, this case suggests that a non-operative approach can be carefully considered in select instances of GAS-associated deep tissue disease. While the discovery of cervical subcutaneous emphysema on CT would normally serve as a strong indication for immediate surgical exploration, several specific clinical factors allowed our team to safely defer surgery. First, despite the presence of emphysema, the CT scan did not reveal extensive gas-forming abscess cavities, fluid collections, or signs of frank tissue liquefaction that would absolutely require mechanical drainage. Second, the patient's airway remained patent and secure, and he remained hemodynamically stable without requiring vasopressor support. Finally, following the rapid escalation to high-dose penicillin G, meropenem, and clindamycin, the patient demonstrated an immediate and progressive stabilization of his vitals and inflammatory markers within hours. By maintaining a high index of clinical suspicion and performing intensive, serial clinical re-evaluations at the bedside, we ensured that surgical intervention remained on standby, ultimately confirming that the deep-tissue infection was being rapidly and effectively controlled by aggressive multi-drug pharmacotherapy alone. While it is well-established that delaying necessary surgery significantly increases mortality [14], our experience suggests that, in highly select, stable patients with soft-tissue infection spectrum disease where irreversible tissue necrosis is absent on prompt imaging, a tightly monitored medical strategy utilizing protein-synthesis-inhibiting antimicrobials can yield an excellent outcome while avoiding the significant morbidity of emergency cervical surgery.

## Conclusions

This case underscores that iGAS infection can present with concurrent cervical necrotizing fasciitis-spectrum involvement and pneumonia, even in young, previously healthy individuals without identifiable predisposing factors. The absence of traditional risk factors should not diminish clinical vigilance for iGAS when patients present with rapid soft-tissue and pulmonary deterioration. Furthermore, while this case demonstrates a successful non-surgical outcome, its generalizability is highly limited, and urgent surgical debridement remains the cornerstone standard of care for definitive necrotizing fasciitis. A conservative strategy should not be widely applied; rather, it represents an exceptional approach that may only be carefully considered in highly select clinical scenarios characterized by the absolute absence of frank necrosis, fluid collections, or abscess formation on prompt imaging, alongside the capacity for continuous, stringent clinical monitoring. When these strict criteria are met, aggressive multi-drug antimicrobial therapy - specifically incorporating toxin-suppressing agents - may allow for successful conservative management while avoiding morbid surgical procedures.
